# Editorial: Sustainable and mission-oriented innovation in economic systems and governance for equitable global health and wellbeing

**DOI:** 10.3389/fpubh.2025.1673178

**Published:** 2025-08-29

**Authors:** Sam Agatre Okuonzi, Humphrey Karamagi, Jolem Mwanje

**Affiliations:** ^1^Ministry of Health (Uganda), Kampala, Uganda; ^2^Organisation mondiale de la Sante pour Afrique, Brazzaville, Republic of Congo; ^3^Universidad Central de Nicaragua, Managua, Nicaragua

**Keywords:** sustainable, mission-oriented, innovation, economic systems, governance, equity, health, wellbeing

The Research Topic “*Sustainable and Mission-oriented Innovation in Economic Systems and Governance for Equitable Global Health and Wellbeing*” explores different approaches to initiate and drive sustainable, mission-led innovations in economic, social, and governance systems. It reflects the commitment to enhance the lives of all people and to protect the environment. The goal is to urge governments and international organizations to innovate systems and to guide new technologies to respond to pressing global challenges that include health insecurity, climate change, population changes, unemployment, mass migration, environmental degradation, and terrorism. It highlights the necessity for a paradigm shift from an economic system focused on maximizing profit for some to one that prioritizes human wellbeing, measured by factors beyond Gross Domestic Product (GDP), such as happiness, healthy lives, social support, and good governance.

The series emphasizes the need for new economic and governance systems to address four core objectives of: eradicating deprivation, reducing inequality, operating within the environmental carrying capacity, and fostering innovation. This requires a transition of the mindset to be rooted in solidarity, equity, human fulfillment, environmental protection, intergenerational care, market regulation, and prosperity defined by quality of life. Governments are seen as crucial in this transformation to actively focus and spend on people. In particular, they are to implement expansionary macroeconomic policies, commit to employment, invest in public works, support low-carbon energy initiatives, and regulate shadow banking.

The Research Topic has generated new ideas, concepts, and policy frameworks, raising awareness and stimulating further discussion on a new sustainable and mission-based economic and governance system.

Studies examined complex interlinks between globalization, economy, and population health, defining globalization as the flow of goods, finance, and ideas across national borders within a country's “economic complexity”. Economic complexity is the totality of human, infrastructure, institutions and organizations that a country uses to produce for export and to distribute income internally. This research suggests that current development models contribute to inequality. To address these development gaps, frameworks were proposed for high-income countries to focus on technology diffusion, middle-income countries on technological innovation and economic transformation, and low-income countries on improving basic services, selective foreign technology use, and cultivating local innovation.

Several studies on costs, cost-effectiveness and willingness to pay for health care highlighted the difficult balance between costs, effectiveness, and willingness to pay (WTP) in healthcare decision-making. For instance, one study recommended Efafirenz combination as a first-line drug for its superior effectiveness despite being slightly more costly. Another found that Trifluridine/tipiracil combined with bevacizumab was not cost-effective as a third-line treatment for colorectal cancer compared to monotherapy. Studies on stroke costs varied significantly by type and health facility level, leading to a recommendation for larger health insurance reimbursement ratios and cost control in private and tertiary hospitals. For progressive glioblastoma, a study found bevacizumab (BEV) with lomustine (LOM) not cost-effective as a first-line therapy, yet acknowledged its survival advantages of offering a treatment option for this rare disease. The dynamic interlinks between health and economic development were exemplified by a study on medical insurance compensation, which views health as a production function through labor and as capital in the economic utility function. This study proposes dynamic medical insurance policies responsive to economic status, requiring constant revision of coverage, fiscal budgets, and healthcare pricing. It also suggests that medical insurance compensation policy should be integrated into existing healthcare systems, with adaptive policy innovations for diverse populations. Factors influencing healthcare expenditure in China were identified as economic development, the aging population, and policy interventions like increased fiscal spending, investment in science and technology, and pandemics management.

The concept of New Type Urbanization (NTU) was analyzed, particularly in China, focusing on coordinated socio-economic and environmental transformation aimed at optimizing economic upgrading, urban spatial structuring, urban-rural integration, and environmental urbanization. NTU links health-related interventions such as improved healthcare services, residences, public transport, and energy use. Issues like population agglomeration within NTU require enhanced information systems, infrastructure upgrading, and investment in health, leisure, and fitness facilities. A related study on industrial structuring emphasized the need for platforms, industrial clusters, collaboration, and investment in local talent for high-quality development. The role of media in reinforcing Environmental, Social, and Governance (ESG) principles was explored, noting its impact on company stock prices, firm value, and reputation. Positive media coverage can reduce the cost of equity, while transparency reports on carbon footprints, social initiatives, labor rights, and diversity efforts are crucial.

Digital Inclusion Finance (DIF), integrating Big Data, Blockchain, and digital technology with the financial industry, was found to catalyze economic growth, particularly promoting research and development (R&D) in pharmaceutical firms. The study recommended government support for DIF, R&D incentives, intellectual property protection, and integration of financial institutions with AI, cloud computing, and new technologies. Another study highlighted the trend of coupling digital economy with older adult care, driven by information and communication systems, local digital innovation, and industrial application of the digital economy. Digital governance was identified as critical for green development, using big data and digital platforms for industrial policy review, financial support, and green projects. The impact of technology mergers and acquisitions on company performance was found to be positive, mediated by R&D, with recommendations for government encouragement and enterprise focus on R&D and skilled labor.

A study on the evolution and driving factors of coupling digital economy with older adult care found this kind of coupling to be a trending pattern. It is associated with densely populated areas of a high income country. The factors that drive digital and older care coupling include availability of information and communication systems, innovation of local digital systems and industrial application of the digital economy. The digital economy of the older adult care is made possible by the availability of service infrastructure, capacity and organization.

Digital governance is critical in digital transformation of a country. Digital governance can be used to promote and sustain green development by supererogation and rationalization of industrial structuring. Digital governance policies must focus on promoting public wellbeing, uplifting less developed cities, and fostering cross-regional cooperation. A study found that digital governance played a key role in industrial policy review, financial support, and entrepreneurship of green industry. Big data was used to asses market demand for products of green industry. Big data, IOT and digital platforms were used for green projects and research.

Competition among enterprises, including pharmaceutical companies, is very high. Technology mergers and acquisitions have now emerged as a key feature of this competition. Pharmaceutical companies improve innovation by internal research and development (which is slow), and by mergers and acquisitions (which is now the preferred method). This amplifies the capacity and ability of the companies to innovate, expand and improve. The purpose of technology mergers and acquisitions is to address deficiencies, diversify and lay foundation for technology innovation.

A study found a positive impact of technology mergers and acquisitions on the performance of companies. Research and development was found to be the mediating factor in the technology performance. However, levels of performance were determined by the amount of financing, size of the firm, and geographical location. Governments need to encourage technology mergers and acquisitions, and provide incentives and guidance. Enterprises must focus on improving research and development, hiring and improving the welfare of highly skilled labor, and in investing in long term training.

Essential Public Health Functions (EPHFs) have been defined as the fundamental, interlinked and interdependent health supporting activities within and beyond the health sector. These include governance, resource generation, financing, and service delivery. Common Goods for Health (CGH), on the other hand, have been described as activities that directly support public health. These include community engagement, social participation, health taxes and subsidies, and public health surveillance and monitoring, health protection and promotion, research, health care supplies and technology. A study mapped EPHFs and CGHs under Health System Performance Analysis (HSPA) framework. The framework provides a visual map of how EPHFs and CGHs are linked to health objectives and goals. The health objectives are listed as quality, equity, access, user expectation, effectiveness, efficiency of services. The final goals are seen as health improvement and protection, and people centeredness.

A study in Eastern China found that both health resource allocation and economic development increased yearly, driven by human and material resources for health, and by the development level and technological investment in the economy. Economic development catalyzes healthcare advancement, and healthcare investment forms a foundation for economic development by providing a healthy workforce. Recommendations included restructuring the health sector to overcome redundancy and inequity, and focusing on primary healthcare at the grassroots level. China's Strategic Health Plan 2030, built on principles of prioritizing health, innovation, coordination, sustainability, openness, and shared development, was highlighted. Investing in health care is also a foundation for economic development by providing a health workforce. Industrial structuring, investment in science and technology, human resources, and health care materials all have positive impact on coordinated health care and economic development.

Rapid economic development, such as in China, is accompanied by increased use of fossil energy and emission of PM2.5—gaseous pollutant droplets or particles that are 2.5 micrometers or less in diameter. These particles are generated by factories, motor vehicles and mechanized agricultural activities. PM2.5 affects human health and the environment. In humans, PM2.5 cause cardiovascular diseases, chronic obstructive pulmonary diseases, cancer and cognitive impairment. On the environment, the particles reduce visibility, cause climate change, and harm ecosystems and wild life. China is transforming from manufacturing to service industry, and is redistributing manufacturing industries. China has instituted broad policies are coordinate regional pollution control, promote clean energy, encourage green/low carbon living.

A study of 27 EU countries found that higher GDP, efficient healthcare spending, and hospital bed availability positively impacted older adult health, while unemployment and inflation had negative effects. This underscored the need for integrated economic and healthcare policies to enhance economic stability, healthcare infrastructure, and chronic disease management for sustainable aging. There was need for strengthening primary health care systems, and for supporting health care workforce. Economic and health care policies would need to be integrated for the welfare of older persons. There is need to investigate sub-populations of older persons vis a vis health care and economic changes.

Healthy aging has been defined as the maintenance of functional abilities in older persons. An older person is 60 years or more. In a study to analyze physical activity and economic autonomy, physical activity was found to be positively associated with economic autonomy for low and moderate physical activities. Physical activity is a health policy matter in aging. It improves mental health, reduces muscle loss and fatal falls, and slows down aging. Economic autonomy was defined as an individual or couple living alone or by themselves. They are able to get a wage and/or pension, they may sell some produce or are self-employed or doing business, for a living. They can afford rent, food and clothing.

An autonomous family is a family with an income that exceeds household expenses. A person who is non-autonomous is involved in high intensity physical activity that is usually a survival activity. Such a person requires government's support. Governments need to make appropriate policy on physical activity by category and economic autonomy status. Such policies should be tailored to local culture taking into account filial piety for older persons and social networks.

National fitness public service is an important aspect in China. However, the efficiency of expenditure of this service was doubtful. A study found the efficiency of spending low at 0.62, with wide disparities among provinces. When policies were introduced, the efficiency rose to 0.76. Redundant expenditures were discovered. There was lack of scientific planning and demand-driven feed back. Collaborative supply mechanisms, integrated technology innovations and accelerated digitization of fitness services were recommended. It was also recommended that private sector be involved through a build-and-operate (BOT) basis.

Life expectancy, which is the average lifespan, is increasing around the world. The difference between the highest and lowest life expectancies is reducing. The increase in life expectancy has been as a result of improved living standards, progress in amenities, health care, education and lifestyle. A study analyzed the factors driving life expectancy with a focus on social expenditure, education expenditure, and real GDP. Social expenditures were defined as cash benefits, tax deductions, goods and services. This study found that the main drivers of life expectancy were health spending and income. Health spending was particularly critical at birth and for the under-fives, and after 60 years. Health expenditure consists of health infrastructure and services, nutrition, immunization, water and sanitation and handwashing.

Health inequality and financial hardship are global challenges. These challenges are much higher for people in the low income group. Rapid economic growth, such as in China, can generate significant health inequality. In China, persons below 67% of median income are categorized as low income group. A study found that self-rated ill-health and chronic disease are much higher in the low income group. Per capita increase of household income reduces the impact of disease and health inequality. Age has a U-shaped effect on health inequality, being higher at young and old ages. The pattern of the effect of low income on health inequality is similar across urban and rural areas, and genders. However, health inequalities are particularly more in older adults and rural groups. It is recommended that low income groups be given income support, be facilitated to access health care, be supported in a family care set-up, and various measures be tailored to the various unique circumstances.

An element of financial hardship has been described in a study as Financial Toxicity (FT). FT is associated with an individual's economic status, the stage of disease and treatment. Factors associated with FT included young age, low education, being uninsured, and low income. Clinicians and health care managers need to identify and conduct psychological interventions as early as possible on people at risk of FT. There is need for financial screening, counseling and referral services. Cancer management, for example, requires health care professionals fostering strong support and collaboration as multi-disciplinary teams. There is need to identify risk factors of FT and explore the interaction between FT and distress.

Factors responsible for medical impoverishment—defined as financial hardship due to healthcare expenses—include unemployment, poor health, chronic disease, small household size, and migrant status. Recommendations focused on expanding health insurance for the poor, poverty alleviation strategies, and social security. A study on rural financial participation, involving formal and informal finance, found that to alleviate rural poverty, people needed to be facilitated to make land transfers and to get non-farm employment, to contribute to common prosperity. Regulation of informal finance and government guarantees for financial stability and infrastructure development were recommended.

Common prosperity among citizens provides a pathway to social equity, sustainable economic growth and long term security. This can be achieved through rural financial participation. A study defined absolute poverty as a person or family whose income could not cover basic necessities of bodily functions. Relative poverty was seen as the exclusion and deprivation in health, education and quality of life. Factors of poverty were identified as low income, inadequate living environment such as housing, consumer goods, living standards, and subjective attitude. Financial participation was found to alleviate poverty. However, there was need for tailored support to non-formal participants with multi-dimensional poverty. Financial participation has to be accompanied by government guarantees to enhance financial stability and infrastructure development.

Studies on integrating medical insurance to be equitable for urban and rural dwellers in China showed increased happiness among returning migrant workers due to improved health insurance systems and equalization of basic health insurance benefits. This achieved the health insurance principle of transferring funds from the healthy to the unhealthy and from the rich to the poor. However, health service use among migrant populations was still found to be highly inequitable, with disparities linked to age, low education, and regional policy variations. Recommendations included health education for migrant communities, increased resource allocation to primary healthcare, and cross-sector collaboration. Health equity is the vital benchmark for gauging the efficacy of health equity policy, which is the safeguard for vulnerable people.

An aspect of inequity is the co-existence of undernutrition and overnutrition or double burden of disease (DBM) within the same population, household, or individual, and is a major concern. A study in Zimbabwe found a 30% increase in DBM over 5 years, with rural women and children suffering more. Promoting food systems that support balanced diets was recommended. Urban dwellers got better access to health care and a diversity of foods, but were exposed to unhealthy dietary practices and sedentary lifestyles. WHO's double duty actions protocol for multiple forms of malnutrition was recommended for Zimbabwe.

Wars, violence and terrorism are well recognized global challenges that erode progress made in health and wellness. Transnational drug trafficking, political unrest, gang violence, and paramilitarism in Haiti have led to a mental health crisis. A study identified chronic traumatic stress, increased health burden, lack of mental health services, hopelessness, future uncertainty, and multigenerational concerns as key issues. Coping factors included mental stimulation, peer support, music, and faith. Exploring historical healing practices was recommended.

This research series underscores that the pursuit of health and wellness is continuous, with new solutions building upon earlier innovations. The paramount message is the need for integrated policy, planning, and implementation of health into governance, economy, technology, and environment. Human health and wellbeing must be central in all endeavors, with a particular emphasis on equity and protection of the poor. Governments are urged to make substantial investments in four key areas: health and wellbeing, environmental protection (bio-systems, rivers, oceans, forests), urban development (housing, transport, water, sanitation), and decarbonization of energy generation. This holistic approach is critical for achieving sustainable and equitable global health and wellbeing.

